# Editorial: Artificial intelligence for cancer immunotherapy

**DOI:** 10.3389/fimmu.2025.1633422

**Published:** 2025-06-11

**Authors:** Issam El Naqa, Shari Pilon-Thomas, Eric Deutsch

**Affiliations:** ^1^ Department of Machine Learning, Moffitt Cancer Center, Tampa, FL, United States; ^2^ Department of Immunology, Moffitt Cancer Center, Tampa, FL, United States; ^3^ Department of Radiation Oncology, Gustave Roussy, Villejuif, France

**Keywords:** immunotherapy, artificial intelligence, cancer, deep learning, oncology

The use of artificial intelligence (AI) technologies and machine learning (ML) algorithms is experiencing tremendous growth in immunology, assisting in various functions such as identifying inflammatory markers related to different immune diseases and frailty, their potential application in designing next-generation monoclonal antibodies and vaccines, and their use to uncover complex patterns in human immune repertoires. The goal of this Research Topic is to highlight the key role of AI/ML in cancer immunotherapy. Given the wealth of existing immunotherapy data and its multi-modal, heterogeneous nature, AI can play a vital role in navigating through this complexity.

Seven articles are presented in this Research Topic with five original research articles and two reviews. The articles cover topics such as predicting antigen peptide presentation, classifying pulmonary nodules, predicting axillary lymph node metastasis, and differentiating immune checkpoint inhibitor-related pneumonitis from pneumonia. The two review articles discuss the role of AI in predicting melanoma response to immunotherapy and the segmentation of malignant lymph nodes. These articles will be discussed briefly below.

Jian et al. developed a novel machine learning framework for predicting antigen peptide presentation by MHC Class I and II molecules. The developed method, called OnmiMHC, can accurately predict peptide-MHC binding affinities across both MHC-I and MHC-II molecules. Its efficacy was demonstrated in Uterine Corpus Endometrial Carcinoma (UCEC), with the method showing promise as a framework for personalized tumor vaccines. 

Zhan et al. developed a prediction model for the classification of pulmonary nodules based on radiomics from preoperative CT imaging and clinical features. Several machine learning methods were evaluated, with an explainable boosting model (EBM) demonstrating the best performance. The pulmonary nodule imaging-grading reporting system (PNI-GARS) was found to be the best predictive feature in their study. 

Two articles studied the prediction of axillary lymph node metastasis in breast cancer patients. Guo et al. developed a multimodal imaging model from mammograms and MRIs. The best model combined traditional radiomics features with extracted deep learning ones using a multilayer perceptron (MLP). In contrast, Ma et al. used ultrasound images to predict axillary lymph node metastasis. Their best model combined sonogram features (microcalcifications) with radiomics using logistic regression.

Duan et al. developed and validated a nomogram for differentiating immune checkpoint inhibitor-related pneumonitis from pneumonia in patients undergoing immunochemotherapy in a multicenter retrospective study. The nomogram was based on the random forest algorithm. The variables used in the nomogram included: smoking status, prior chronic obstructive pulmonary disease (COPD), ground glass opacities, non-specific interstitial pneumonitis, Neutrophil-to-Lymphocyte Ratio (NLR), pleural effusions, and Oxygen Partial Pressure (PaO2).

A review article on the role of machine learning in predicting melanoma response to immunotherapy was presented by Li et al. The authors conducted a bias assessment on the prediction model risk of bias assessment tool (PROBAST) and a meta-analysis. They studied a total of 36 studies, which included 30 cohort studies and 6 case-control studies. They analyzed the outcome measures of progression-free survival, overall survival, and treatment response. Their findings indicated that there is considerable predictive accuracy in melanoma immunotherapy response and prognosis, however, there was a systematic lack of external validations in many cases.

Finally, a review of deep Learning applications for malignant lymph node segmentation and detection was presented by Wu et al. This contribution is valuable for treatment planning purposes. The review covered five clinical sites - head and neck, upper extremity, chest, abdomen, and pelvis - highlighting current challenges and future trends that would impact clinical application.

These articles, despite their value in highlighting the role of AI in immunotherapy, covered only a fraction of the potential applications of AI/ML in immunotherapy, particularly in areas related to understanding primary or secondary tumor resistance to immunotherapy, identifying improved biomarkers, and developing new treatment strategies to personalize immunotherapy. For instance, AI can identify candidate biomarkers that can be applied clinically to select patients for the appropriate immunotherapy regimens, saving others from unwanted toxicities and costs (1).

This Research Topic highlights the significant impact of AI and ML in transforming cancer immunotherapy. It provides new insights and tools to address the complex challenges in this area. By utilizing advanced computational methods, researchers are improving the accuracy of diagnostic and therapeutic approaches and creating innovative solutions that could change patient care and outcomes. The use of AI and ML in immunotherapy research represents a major shift that promises to accelerate the development of more effective and personalized treatment strategies. The pace of these changes is likely to be tremendous in the years to come.
